# Supplementary figures and images for: A comprehensive prognostic and immunological analysis of hexokinase domain containing protein-1 (HKDC1) in pan-cancer
Source: PeerJ. 2025 Mar 19;13:e19083. doi: 10.7717/peerj.19083 (PMC11929506; doi:10.7717/peerj.19083)

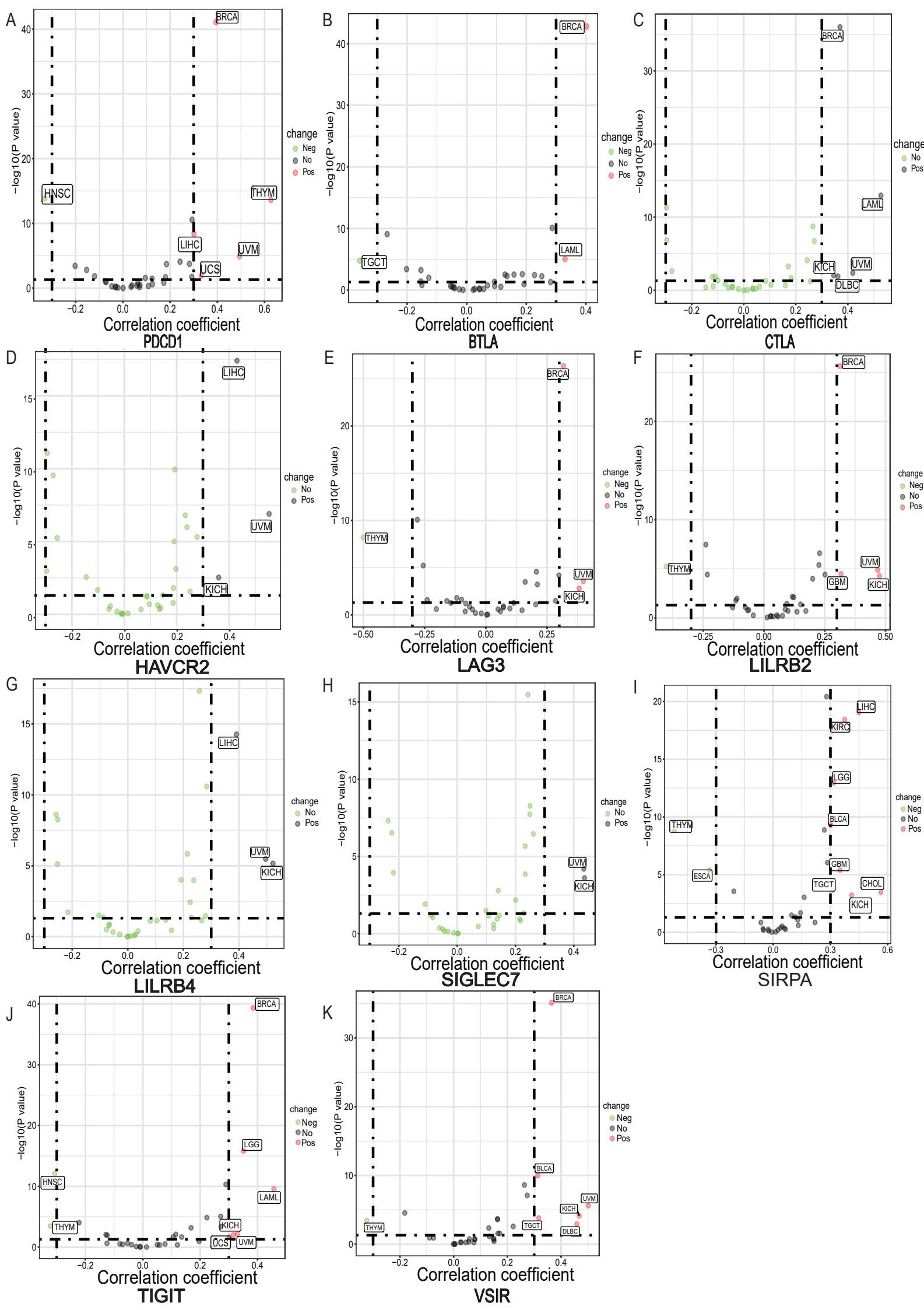

Supplement: Supplemental Information 1 — (A) PDCD1, (B) BTLA, (C) CTLA4, (D) HAVCR2, (E) LAG3, (F) LILRB2, (G) LILRB4, (H) SIGLEC7, (I) SIRPA, (J) TIGIT, and (K) VSIR across various cancer types. [file peerj-13-19083-s001.pdf]

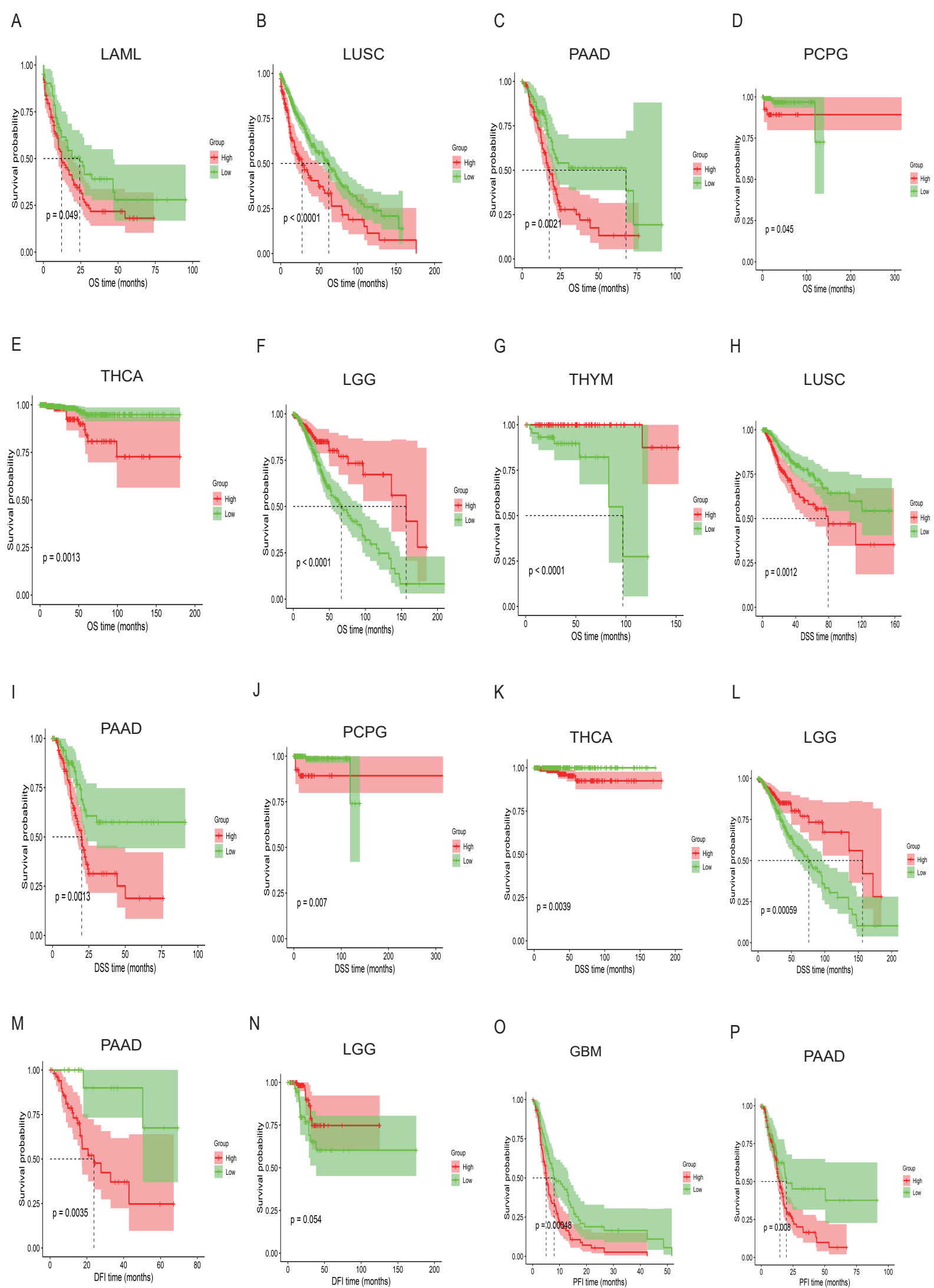

Supplement: Supplemental Information 2 — Additionally, Kaplan-Meier survival curves have been generated to highlight the correlations between HKDC1 mRNA expression and OS, DSS, DFI, and PFI among patients, as depicted in panels (A–P). [file peerj-13-19083-s002.pdf]

Figure7

A

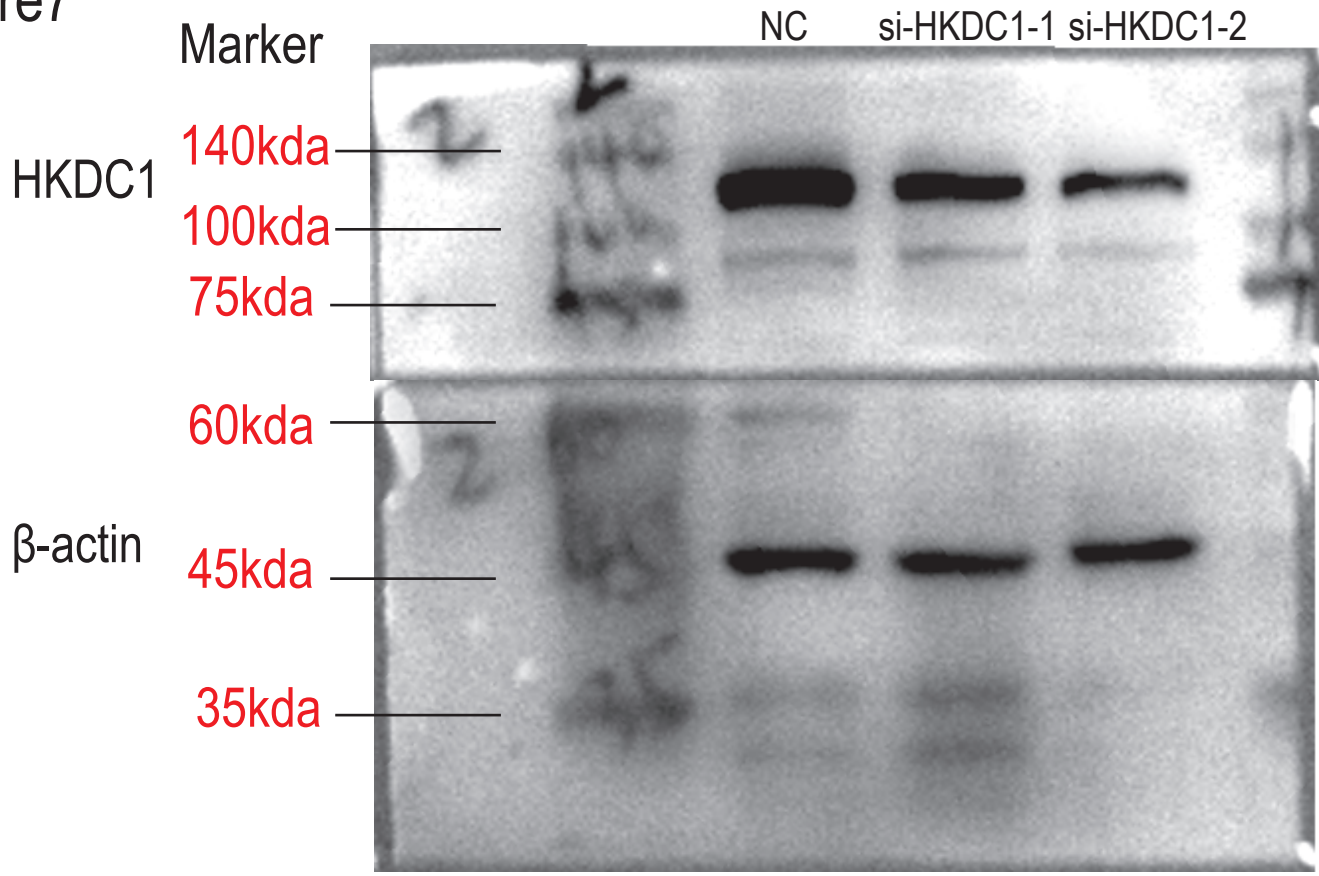

E

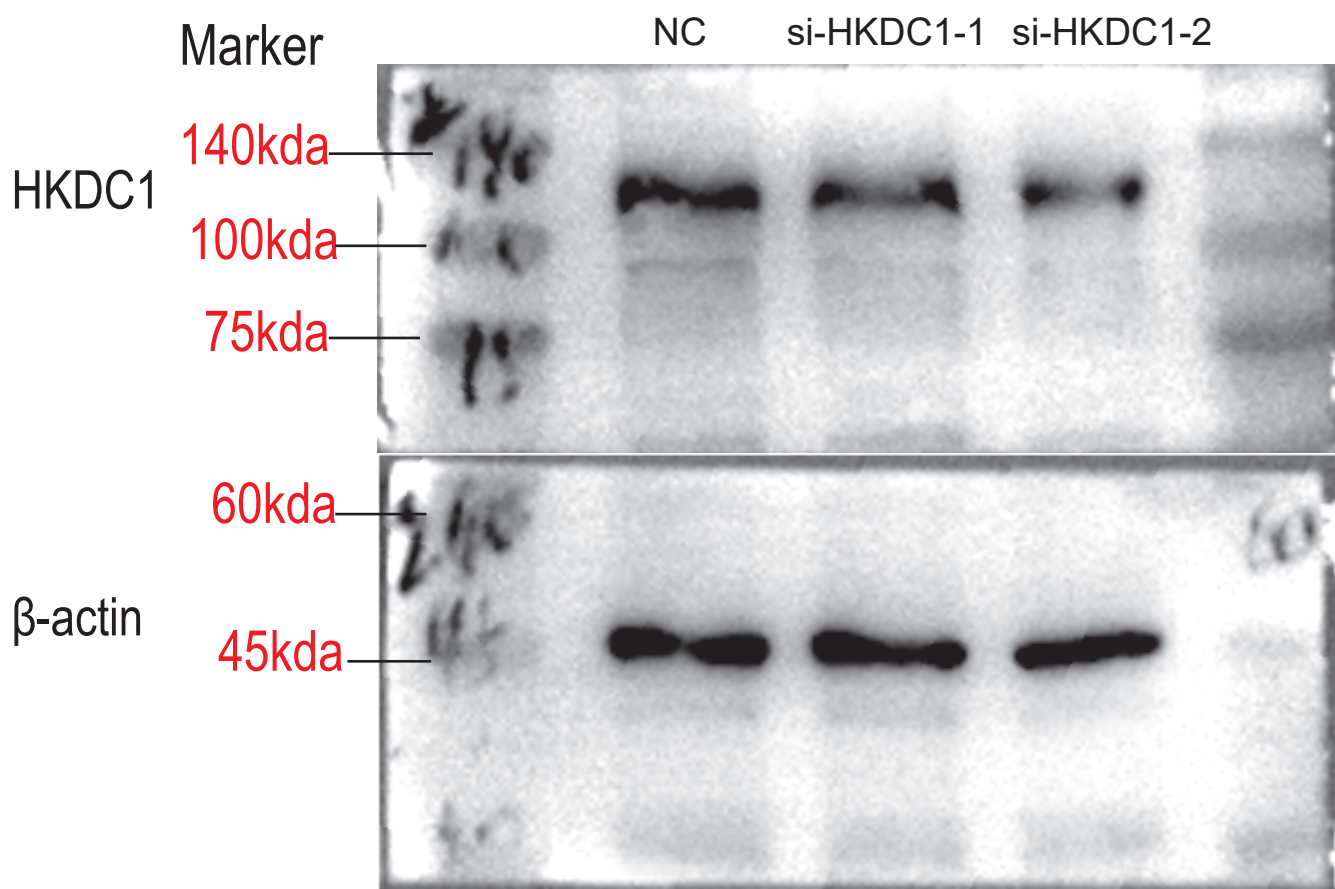

Supplement: Supplemental Information 3 [file peerj-13-19083-s003.pdf]
